# Infantile Diseases Depending upon Inflammation

**Published:** 1846-03

**Authors:** P. Hood

**Affiliations:** Surgeon, &c.


*On the Distinction between Infantile Diseases depending upon Inflammation and those which arise from Irritation.* By P. Hood, Surgeon, &c.—The importance of distinguishing between the symptoms of inflammation and irritation cannot be too strongly urged. Every practitioner must have experienced the disappointment which sometimes attends his best endeavors to relieve a suffering child, and been apprehensive of his remedies, more especially when he has had any doubt as to the cause of the disease. But by bearing in mind that morbid irritation exerts a powerful influence over the diseases of children, then treatment becomes more simple, and greater confidence is acquired. This consideration will in a great measure prevent the very common occurrence of the prostration of the strength of the child, at the onset of its disease, by the use of exhausting and lowering remedies. It will lead the practitioner to observe that children's diseases are not so speedy in their termination as is generally supposed, and that where he has hesitated to abstract blood, and has used more lenient means he has been rewarded; that the child, so far from sinking from want of blood-letting, has not only survived, but has proceeded rapidly to recover its health; while under contrary treatment, if it survived at all, its convalescence has been slow and protracted.

The practice of bleeding and leeching is inadmissible in diseases having their origin in morbid irritation. The only instances in which I have ever observed blood-letting and leeches borne with comparative impunity as remedies for what are termed the inflammatory diseases of childhood, have been at the earliest period of the attack, and when the greatest possible care has been taken to limit the quantity of blood taken. But even in these cases I have observed that the children long looked pale and sickly, and that some of them sunk under a renewal of the disease, to which the treatment adopted appeared to render them more liable.

The treatment most successful in allaying irritation comprises emetics, sedatives, and stimulating embrocations, when the lungs are attacked; sedatives, and aperients of a well-regulated strength, with febrifuge medicines when the brain is the seat of the disorder.—*Ibid.*

